# The Impact of Seasonal and Annual Climate Variations on the Carbon Uptake Capacity of a Deciduous Forest Within the Great Lakes Region of Canada

**DOI:** 10.1029/2019JG005389

**Published:** 2020-09-18

**Authors:** Eric R. Beamesderfer, M. Altaf Arain, Myroslava Khomik, Jason J. Brodeur

**Affiliations:** ^1^ School of Earth, Environment and Society and McMaster Centre for Climate Change McMaster University Hamilton Ontario Canada; ^2^ Geography and Environmental Management University of Waterloo Waterloo Ontario Canada

**Keywords:** carbon, net ecosystem productivity, eddy covariance, deciduous forest, white oak, southern Ontario

## Abstract

In eastern North America, many deciduous forest ecosystems grow at the northernmost extent of their geographical ranges, where climate change could aid or impede their growth. This region experiences frequent extreme weather conditions, allowing us to study the response of these forests to environmental conditions, reflective of future climates. Here we determined the impact of seasonal and annual climate variations and extreme weather events on the carbon (C) uptake capacity of an oak‐dominated forest in southern Ontario, Canada, from 2012 to 2016. We found that changes in meteorology during late May to mid‐July were key in determining the C sink strength of the forest, impacting the seasonal and annual variability of net ecosystem productivity (NEP). Overall, higher temperatures and dry conditions reduced ecosystem respiration (RE) much more than gross ecosystem productivity (GEP), leading to higher NEP. Variability in NEP was primarily driven by changes in RE, rather than GEP. The mean annual GEP, RE, and NEP values at our site during the study were 1,343 ± 85, 1,171 ± 139, and 206 ± 92 g C m^−2^ yr^−1^, respectively. The forest was a C sink even in years that experienced heat and water stresses. Mean annual NEP at our site was within the range of NEP (69–459 g C m^−2^ yr^−1^) observed in similar North American forests from 2012 to 2016. The growth and C sequestration capabilities of our oak‐dominated forest were not adversely impacted by changes in environmental conditions and extreme weather events experienced over the study period.

## Key Points

- Seasonal and annual carbon fluxes were studied from 2012 to 2016 at a mature temperate deciduous forest in southern Ontario, Canada
- Meteorological conditions over a short midsummer period greatly impacted annual NEP
- On average, the forest was an annual carbon sink of 206 ± 92 g C m^−2^ yr^−1^

## 1 Introduction

Temperate forests occupy nearly 25% of the global forested land area (~10.4 million km^2^), primarily in eastern North America, Europe, and Eastern Asia (Settele et al., 2014; Tyrrell et al., 2012). They store roughly 99 to 159 Pg of C (~11% of the global C stock) and account for up to 37% of the global forest C sink (Pan et al., 2011). Most temperate forests are deciduous stands, which cover an area of 7.8 million km^2^ across the world (Allaby, 2006; Vasseur, 2012). In eastern North America, temperate deciduous forests covered about 2.5 million km^2^ of land area at their peak coverage in the eighteenth century and were considered a dominant forest cover type (Botkin et al., 1993; Delcourt & Delcourt, 2000). These forests have been severely impacted by human activities over the last 100 yr (Gao et al., 2012; Johnston, 2009). Widespread deforestation occurred during the early 1900s in eastern North America for agricultural purposes, causing a drastic reduction in forest cover from 90% to 11% (Richart & Hewitt, 2008). Later in the mid‐1900s, abandonment of agricultural lands and forest regrowth initiatives led by the local governments resulted in an increase of forested area (Hansen et al., 2013; Richart & Hewitt, 2008). Currently, secondary growth forests occupy 40% to 50% of their original cover in some regions of eastern North America (Tyrrell et al., 2012). Long‐term regrowth of these secondary growth forests has helped North America to become a major C sink (Birdsey et al., 2006). However, there are uncertainties about the strength and resiliency of this C sink under future climate change; a topic of discussion in recent literature (Curtis & Gough, 2018).

The seasonal cycles of C fluxes in temperate deciduous forests are unique, as in these forests, foliage must first be produced in order for photosynthetic processes to take place. This leads to a shortened growing season each year, where ecosystem respiration (RE) is the dominant C flux over the first 4–5 months of the year (Goulden et al., 1996 *;* Greco & Baldocchi, 1996 *;* Richardson et al., 2010). While photosynthesis begins much later in deciduous forests compared to evergreen coniferous forests, the photosynthetic C uptake rates of deciduous forests are much higher, sometimes twice as high as coniferous stands (Gaumont‐Guay et al., 2009). Because of the shorter growing seasons, temperate deciduous forests are potentially more vulnerable to climate change and extreme weather events (Vose et al., 2012).

Studies in the literature have shown significant changes in the climate of eastern North America, leading to warmer temperatures (particularly in winters) and greater winter and annual precipitation (*P*), affecting both local and regional hydrologic cycles (Iverson et al., 2008; U.S. Global Change Research Program (USGCRP), 2017; Zhu et al., 2012). In the Great Lakes region specifically, air temperature (Ta) has increased by 0.89°C, while *P* has increased by 10% over the past 114 yr (1901–2015) (IPCC, 2013; USGCRP, 2017). Apart from increasing Ta, future climate change is expected to alter the distribution of *P* over the year in this region, with expected summer water deficits of 5–10% by the end of the century (Byun & Hamlet, 2018; O'Gorman & Schneider, 2009). This area is also expected to experience longer growing seasons due to shorter and warmer winters, which in turn may perturb the balance between gross ecosystem productivity (GEP) and RE (Barr et al., 2002; Byun et al., 2018). Additionally, while warmer winter temperatures and an earlier start to the growing season may enhance C uptake in spring, summer droughts and warming in the late growing season or autumn could enhance respiratory losses, offsetting gains from the extended period of photosynthetic uptake (Froelich et al., 2015; Piao et al., 2008; Richardson et al., 2010; Schimel, 1995). Over the longer term, these variations in environmental conditions may also lead to changes in leaf phenology (leaf emergence and senescence) or stand composition, which could greatly impact the C uptake capacity of eastern North American forest ecosystems, particularly those within the Great Lakes region (Aerts, 1995; Curtis et al., 2002; Fisichelli et al., 2013; Froelich et al., 2015; Malhi et al., 1999; Way & Oren, 2010; Wilson & Baldocchi, 2000). Therefore, there is an urgent need to conduct long‐term observational studies which look to explore the responses of temperate deciduous forests to climate change in this biologically and economically important region of North America.

The main goal of this study was to evaluate the impacts of climatic variability and extreme weather events on the C sink‐source capacity of a temperate deciduous forest growing in the Great Lakes region of North America. The specific objectives were to (i) continuously measure CO_2_ fluxes and meteorological and soil variables from 2012 to 2016, (ii) determine key environmental controls on the seasonal and interannual variability of net ecosystem productivity (NEP), and (iii) compare the growth and productivity of this forest to other similar deciduous forests across eastern North America.

## 2 Methods

### 2.1 Site Description

The study site (42°38′7″N, 80° 33′28″W; elevation 265 m) is located north of Lake Erie near Long Point Provincial Park, roughly 5 km southwest of Walsingham in Norfolk County, Ontario, Canada. This ecoregion of temperate forests is also known as Carolinian forests, which range from southeastern Canada in the north to the Carolinas in the United States to the south (Blouin*,* 2001 *;* Johnston, 2009; Lauriault*,* 1989; Solomon & Bartlein*,* 1992). Forests in the area cover roughly 18% to 25% of the land surface within the agricultural landscape. The study site is part of the Turkey Point Observatory and is known as Canadian Turkey Point Deciduous Forest (CA‐TPD) in the global FLUXNET network. The Turkey Point Observatory is composed of an age sequence of three planted and managed white pine conifer forests (Peichl et al., 2010) and this 70‐ to 110‐yr‐old, naturally regenerated deciduous forest. The forest is growing on abandoned agricultural land and has been subject to periodic timber extraction. No management activity has occurred in the forest since 1994 (Long Point Region Conservation Authority [LPRCA] records). The site is owned by the Ontario Ministry of Natural Resources and Forestry (OMNRF) and managed by the LPRCA (Parsaud, 2013).

The site is predominantly composed of hardwood species with a few scattered conifer species. White oak (*Quercus alba*) is the dominant tree species, while other tree species include sugar (*Acer saccharum*) and red (*Acer rubrum*) maple, American beech (*Fagus grandifolia*), black (*Quercus velutina*) and red (*Quercus rubra*) oak, and white ash (*Fraxinus americana*), with white pine (*Pinus strobus*) comprising roughly 5% of the forest's tree population. A sample of tree cores taken on site date the oak species back to 1942, while some pine species appear to have begun growing around 1903. The extensive understory is made up of young deciduous trees as well as other plants including Canada mayflower (*Maianthemum canadense)*, putty root (*Aplectrum hyemale*), yellow mandarin (*Disporum lanuginosum*), red trillium (*Trillium erectum*), horsetail (*Equisetum*), and other species. The forest is rich in biodiversity with a total of 573 tree and plant species (Elliott et al., 1999).

Biometric measurements were conducted in 2012 following Canadian National Forestry Inventory (NFI) protocol (Kula, 2013; Parsaud, 2013). The data revealed that the mean tree height was 25.7 m, while the mean tree diameter at breast height (DBH = 1.3 m) was 23.1 cm. The mean stand basal area was 21.2 m^2^ ha^−1^_,_ the mean stand density was 504 ± 181 trees ha^−1^, and the stand volume was 381.4 m^3^ ha^−1^. In 2012, the total C stored in aboveground biomass was 83.10 t C ha^−1^ (Kula, 2013). The maximum leaf area index (LAI) measured in 2012 using a plant canopy analyzer (LAI‐2000, LI‐COR Inc.) and TRAC (Tracing Radiation and Architecture of Canopy, following Chen*,* 1996) was 8.0 m^2^ m^−2^. Site characteristics are summarized in Table 1.

**Table 1:** Site Characteristics

Stand parameter	Condition
Location	42°38′7.124″N
	80°33′27.222″W
Stand age	70–110 yr
Elevation above sea level	265 m
Dominant overstory species	White oak (*Quercus alba*)
Secondary overstory species	Red oak (*Quercus rubra*)
	Sugar maple (*Acer saccharum*)
	White pine (*Pinus strobus*)
	Red maple (*Acer rubrum*)
	American beech (*Fagus grandifolia*)
	Yellow birch (*Betula alleghaniensis*)
Understory species	Putty root (*Aplectrum hyemale*)
	Yellow mandarin (*Disporum lanuginosum)*
	Canada mayflower (*Maianthemum canadense)*
	Red trillium (*Trillium erectum*)
	Black cherry (*Prunus serotina*)
	Wood violet (*Viola palmata*)
	Horsetail (*Equisetum*)
Maximum leaf area index (LAI)	8.0 m^2^ m^−2^
Mean diameter at breast height (DBH)^a^	23.14 ± 14.05 cm
Mean tree height^a^	25.7 ± 7.44 m
Stem density^a^	504 ± 181 ha^−1^
Mean tree basal area^a^	0.06 ± 0.02 m^2^

^**a**^ Kula (2013).

The site is located within the Southern Norfolk Sand Plains, an area defined by coarse‐grained, sandy deposits from past ice age glacial melt processes (Richart & Hewitt, 2008). The sandy soils are well drained with a low‐to‐moderate water holding capacity, and are defined by the Canadian Soil Classification Scheme as Brunisolic Gray‐Brown Luvisol (Presant & Acton*,* 1984). A regional soil analysis described similar soils in the area as having a 20 cm thick Ap horizon, followed by a 30 cm Bm1 horizon, and a Bm2 horizon reaching up to 80 cm (Presant & Acton, 1984). Site measurements in 2012 found a 5–10 cm thick litter layer and an organic‐rich loamy‐sand layer (18% organic matter), while lower soil layers were composed of over 90% sand and contained <2% organic matter. Additionally, the average bulk density of the sand was estimated to be 1.15 g cm^−3^, with an average soil pH of 5 (Parsaud, 2013).

The climate of the region is humid temperate with warm, humid summers and cool winters (Environment and Climate Change Canada (ECCC), 2019). The moderating effect of nearby Lake Erie helps to regulate cold winter temperatures. ECCC records from the ECCC Delhi CDA weather station (25 km north of the site) found that on average (during the period from 1981–2010), the area experienced 145 days of frost‐free weather, a mean annual air temperature of 8.0 ± 1.6°C and 997 mm of total mean annual precipitation. Precipitation is evenly distributed throughout the year, with 13% of that falling as snow.

### 2.2 Eddy Covariance Flux Measurements

Half‐hourly fluxes of momentum, latent heat (LE), sensible heat (*H*), and CO_2_ (*F*_c_) were measured continuously using a closed‐path eddy covariance (EC) system (CPEC) from 2012 to 2016. The CPEC consisted of an enclosed infrared gas analyzer (IRGA: LI‐7200, LI‐COR Inc.) and a 3‐D sonic anemometer (CSAT3, Campbell Scientific Inc.). The IRGA and CSAT3 were mounted at 36 m height atop a walk‐up scaffold tower. The CSAT3 was installed and oriented facing west (270°), while a 1 m long intake tube immediately behind the CSAT3 delivered sampled air to the IRGA. A flow module (7200‐101, LI‐COR Inc.) helped to regulate and control the air flow rate through the IRGA. Air was drawn at a flow rate of 15 L/min, maintaining turbulent flow. The IRGA was calibrated once a month using high purity nitrogen gas for the zero offset, and ECCC Greenhouse gas laboratory specified CO_2_ gas concentrations for the CO_2_ span check. Midcanopy CO_2_ concentrations were measured at 16 m height using a second IRGA (LI‐820, LI‐COR Inc.) in order to calculate the two‐level CO_2_ storage fluxes, as described below. Fluxes were measured at 20 Hz frequency and averaged to half‐hourly values using a custom software created by the Biometeorology and Soil Physics Group at the University of British Columbia, installed on a desktop computer housed in a trailer at the site.

The CO_2_ storage (*S*_CO2_) within the air column below the EC sensors was calculated by vertically integrating the difference between the current and previous half‐hourly values of CO_2_ concentrations measured above the canopy at 36 m height and at the midcanopy level at 16 m height. At times when midcanopy measurements were not available, the change in storage was calculated from the above‐canopy measurements only. Morgenstern et al. (2004) showed there to be good agreement (within 10%) between four‐level storage flux calculations and a single above‐canopy calculation of the storage flux in a Douglas fir forest in Canada. Net ecosystem exchange (NEE, μmol m^−2^ s^−1^) was calculated as the sum of the vertical CO_2_ flux (*F*_c_), and the change of CO_2_ storage (NEE = *F*_c_ + *S*_CO2_). Vertical and horizontal advections were assumed to average to zero over long periods and thus were not considered. NEP was calculated as the inverse of NEE (NEP = −NEE), where positive NEP indicates net carbon uptake by the forest (sink), and negative NEP is carbon loss from the forest to the atmosphere (source).

### 2.3 Meteorological Measurements

Meteorological measurements were also made simultaneously with EC measurements since January 2012. Relative humidity (RH) and Ta were measured (HMP155A, Campbell Scientific Inc.) at 36 m height. A compact aspirated shield (43502‐L, R.M. Young Company) was used to mount the sensor, providing continuous ambient air over the sensor head, while also protecting it from solar radiation interference. Both wind speed and direction were recorded in the first three years of measurements using a Model 85000 anemometer (R.M. Young Company) that was replaced (Model 05103, R.M. Young Company) in 2015. Upward and downward photosynthetically active radiation (PAR, PQS1, Kipp and Zonen B.V.) and all four components of radiation (CNR4, Kipp and Zonen B.V.) were also measured at 36 m height.

Atmospheric pressure (61302V, R.M. Young Company), and snow depth (model SR50A, Campbell Scientific Inc.) were measured at the ground. Precipitation (*P*) was measured in a small forest opening 350 m southwest of the tower, using an all‐season, heated tipping‐bucket rain gauge (model CS700H, Campbell Scientific Inc.). The rain gauge was installed at 1.5 m height and was protected by an Alter Wind Screen (260‐953, Campbell Scientific Inc.). Precipitation data were cross‐checked and occasionally gap filled from an accumulation rain gauge data (T‐200B, GEONOR Inc.), installed 15 km east, near the 80‐ and 45‐yr‐old conifer sites of the Turkey Point Observatory. Soil temperature (Ts) and soil water content (*θ*) were measured using temperature (model 107, Campbell Scientific Inc.) and moisture probes (model CS650, Campbell Scientific Inc.) at 2, 5, 10, 20, 50, and 100 cm depth in two different locations. Soil heat flux (G) measurements were made using four soil heat flux plates (model HFT3, Campbell Scientific Inc.) buried 3 cm below the surface at two locations. Meteorological, soil, and precipitation data were sampled and recorded using multiple data loggers (CR3000 and CR10X, Campbell Scientific Inc.). Automated data downloads were conducted every half hour using the desktop computer.

### 2.4 Leaf Phenology Measurements

Phenological imagery was acquired at half‐hourly temporal resolution using a PhenoCam (Richardson et al., 2007, 2009) installed facing north on top of the flux tower at 36 m height. The start and end dates of the growing season were estimated using phenological transition dates (*phenophases*) calculated by the greenness chromatic coordinate (GCC) derived by the PhenoCam. From the GCC minima and maxima, dates identifying the 10%, 25%, and 50% amplitude of greenness rising and greenness falling stages were then identified (Richardson et al., 2018). A range of transition dates (10–50%) were used in this analysis to identify the start and end of the growing season. All GCC data retrieval and postprocessing analysis of the PhenoCam transition dates was done using the PHENOCAMR R package (Hufkens et al., 2018).

These dates were only used for the purpose of identifying key seasonal transition periods within this study. Seasons were defined as spring (March, April, and May), summer (June, July, and August), fall (September, October, and November), and winter (December, January, and February).

### 2.5 Data Processing, Gap‐Filling, and Statistical Analysis

The flux and meteorological data were filtered, cleaned (threshold and point cleaned), and gap filled using the Biometeorological Analysis, Collection, and Organizational Node (BACON) software following protocols designed by the AmeriFlux Network (Brodeur, 2014). Outliers in the data were identified and removed. Small gaps (a few hours) within meteorological data were linearly interpolated, while larger gaps (hours to days) were filled using linear regression model fitted values from other Turkey Point Observatory sites. Overall, the mean flux recovery was 89% (ranging from 83% to 94%) over the 5 yr of the study.

A footprint model, following Kljun et al. (2004), was applied to exclude fluxes when greater than 10% of the flux footprint extended outside of the forest boundary. During periods of low turbulence, typically at night within a stable nocturnal boundary layer, EC measurements may underestimate fluxes. To resolve this underestimation, threshold passing methods were incorporated in the data processing after the footprint analysis. To remove unrepresentative measurements, a friction‐velocity, u‐star threshold (*u* ^*Th^), was applied to all nocturnal NEE measurements (Barr et al., 2013; Papale et al., 2006). Half‐hourly NEE values were removed from the data set when the measured *u* ^*^ was below thresholds estimated using the Moving Point Test *u* ^*Th^ determination method (Reichstein et al., 2005). This method estimated *u* ^*Th^ from the relationship between nighttime NEE and *u* ^*^ (Papale et al., 2006). An average, site‐specific, *u* ^*Th^ of 0.40 m s^−1^ was determined, where nighttime NEE values below this threshold were removed. These data were filled using exponential relationships between sufficiently turbulent (*u* ^*^ > 0.4 m s^−1^) nighttime NEE and Ts at 5 cm depth. Following the aforementioned threshold passing methods, the mean capture for non‐gap‐filled NEE was 49% (from 46% to 53%) annually.

The partitioning of NEE into components of RE and GEP was achieved using methods described in Peichl et al. (2010). RE was assumed to be equivalent to NEE during the nighttime (PAR < 100 μmol m^−2^ s^−1^). These values were used to model a continuous RE timeseries as a function of Ts_5cm_ and *θ*_0‐30cm_ (Brodeur, 2014) using fitted temperature response parameters (*R*_10_ and *Q*_10_) to describe the relationship between RE and Ts_5cm_, modified by a soil moisture function:

(1) $$\mathrm{RE}=R_{10}\times Q_{10}^{\frac{\left(\;{\mathrm{Ts}}_{5\;\mathrm{cm}}-10\right)}{10}}\times \frac{1}{\left[1+\exp\left(a_{1}-a_{2}\;\theta_{0-30\;\mathrm{cm}}\right)\right]}$$

where *a*_1_ and *a*_2_ are fitted parameters as a function of the independent variable, *θ*_0‐30cm_, acting to scale the Ts_5cm_‐RE relationship. The half‐hourly GEP was estimated by adding measured and footprint‐filtered NEP and modeled daytime RE. Gaps in the GEP timeseries were modeled using a rectangular hyperbolic function:

(2) $$\mathrm{GEP}\;=\;\frac{\alpha \text{PARd}\;A_{\max}}{\alpha \text{PARd}+A_{\max}}\;\times ʄ\left(T_{s}\right)\times ʄ\left(\mathrm{VPD}\right)\times ʄ\left(\theta_{0-30\mathrm{cm}}\right)$$

The first term defines a relationship between PAR and GEP. The remaining terms are scaling responses of GEP to *T*_s_, vapor pressure deficit (VPD), and *θ*_0‐30cm_, respectively (Brodeur, 2014). Where meteorological data were missing to compute RE and GEP, gaps were filled using a nonlinear regression approach and a marginal distribution sampling approach (Brodeur, 2014; Reichstein et al., 2005). Gaps in NEP, due to instrumentation errors, maintenance, calibrations, and power outages, were filled as the difference between the modeled GEP and modeled RE.

Additionally, in order to determine the most significant environmental controls on half‐hourly and daily non‐gap‐filled NEE within the deciduous forest, a multivariate regression model was fit to the data using incident PAR, VPD, Ts_5cm_ and *θ*_0‐30cm_ as explanatory variables (see supporting information Tables S1 and S2. AIC and BIC criterion were used to select the best fit model). These variables were chosen due to their usage in the aforementioned gap‐filling methods. The analysis was restricted to daytime (PAR > 100 μmol m^−2^ s^−1^) growing season data. Variables included in the model were significant at *α* < 0.05, while the model fit was determined using the residual analysis of the coefficient of variation (*R* ^2^). In order to determine the relative contribution or explanatory power of the environmental variables derived from the best fit model, a residual analysis was completed (Lindeman et al., 1980; Skubel et al., 2015). The contribution of each variable to the model *R* ^2^ was determined by the differences in fit between the total model (i.e., all variables) and the model without individual variables, resulting in a percentage decrease of total model *R* ^2^. All data processing and analyses were completed using MatLab software (The MathWorks Inc.).

### 2.6 Regional Analysis

We identified relevant studies in the peer‐reviewed scientific literature of the past 20 yr, which reported annual NEE or NEP values measured by EC systems in North America. The ISI Web of Science was searched for the following specific terms: “eddy covariance”, “net ecosystem productivity”, “eastern deciduous”, “temperate forest”, “North America”. These searches yielded numerous results, highlighting the past research that has been conducted on carbon fluxes within eastern deciduous forests. To be included in our analysis, studies had (1) to report annual NEE values from EC measurements for the majority (3+ yrs) of the 2012 to 2016 period; and (2) the temperate deciduous forest sites needed to be located in North America. In an attempt to expand the study, we also considered sites that reported past peer‐reviewed annual NEE measurments, outside of the study period. In three such cases, the authors were contacted for unpublished data, or were asked to confirm the accuracy of data downloaded from AmeriFlux (http://ameriflux.lbl.gov). The reporting of standard deviations was not essential as flux errors are often not reported in the literature. However, confidence intervals (95%) incorporating the effect of random instrument error and both systematic and random errors associated with the calculation of annual NEE values were estimated for our site. NEE model uncertainty ranged from ±33 to 37 g C m^−2^ yr^−1^ over the study period. Similarly, at a forest included in the regional analysis, the reported uncertainty in NEE ranged from ±30 to 49 g C m^−2^ yr^−1^ for the same period (Moser et al., 2020). Lastly, forest age, annual mean Ta and annual total *P* were taken from the cited study or AmeriFlux database, when not readily available. Ultimately, if no recent data could be found on the Web of Science or validated from external sources, sites were excluded from the study. Consequently, a total of 5 sites (including our forest) encompassing 23 yr of data from 2012 to 2016 were analyzed in this study.

## 3 Results

### 3.1 Environmental Variability

The mean annual Ta between 1 January 2012 and 31 December 2016 was 9.76 ± 1.5°C, at our study site, while the mean total annual *P* for the same measurement period was 881 ± 102 mm. In comparison, the 30‐yr (1981 to 2010) mean Ta and *P* values measured at the nearby ECCC weather station in Delhi, Ontario, were 8.0 ± 1.6°C and 997 ± 145 mm, respectively. The 5 yr mean annual Ta at our site exceeded the 30‐yr mean Ta by 1.7°C, with most years indicating warmer conditions than the 30‐yr mean values. The highest mean annual Ta was in 2012 (11.8°C) and the lowest in 2014 (8.0°C). The highest annual *P* sum was observed in 2014 (991 mm), while the lowest annual *P* was observed in 2015 (750 mm). Three years (2013, 2014, and 2016) had annual *P* values similar to the 30‐yr mean *P*, however none of them exceeded the 30‐yr mean *P* value of 997 mm.

**Figure 1:**
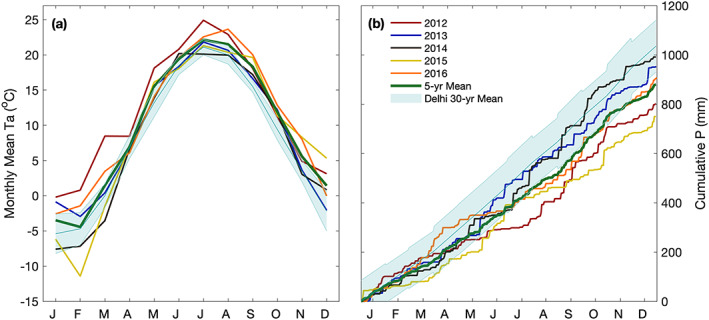
Monthly mean (a) air temperature (Ta) and (b) cumulative precipitation (*P*) from 2012 to 2016. The site‐specific mean values are shown with a green line, while the 30‐yr (1981 to 2010) mean ECCC values are shown in teal with ±1 standard deviation shaded.

**Figure 2:**
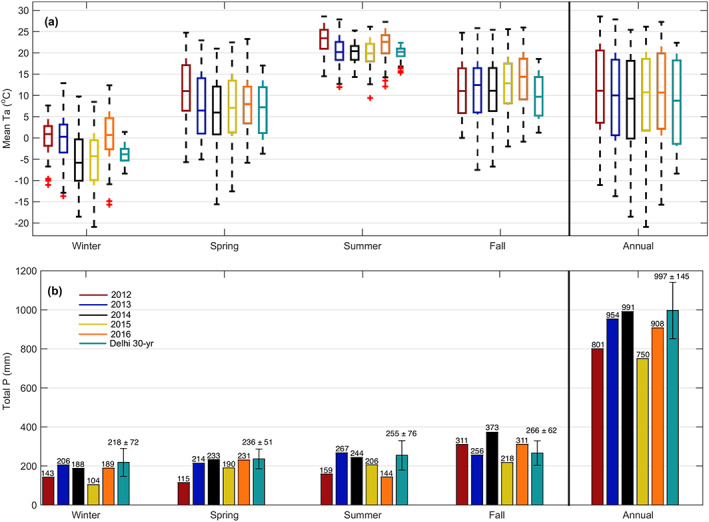
(a) Box plot of mean seasonal and annual air temperature (Ta) and (b) the total seasonal and annual precipitation (*P*) values from 2012 to 2016. The 30‐yr (1981 to 2010) mean ECCC values are also shown in teal. Error bars represent the range of uncertainty.

Figure 1 illustrates the monthly mean Ta and cumulative *P* sum measured at the site. The corresponding 30‐yr mean values are also shown. The Ta followed a similar annual trend over all 5 yr, reaching a minimum in January and February, and a maximum in late summer, primarily in July and August. From April to October the mean monthly temperature remained above 10°C in all years. The coldest month was February 2015 (−11.4°C), however 2015 ended the year with the highest mean December Ta (5.3°C). In 2012, the mean monthly Ta was the warmest of all 5 yr for the period from January through July, leading to a summer drought. Furthermore, late growing season warming was experienced in 2016, with unusually warm mean monthly Ta in August (23.6°C) and September (20°C), which was 3.6°C and 4.5°C above the 30‐yr mean, respectively (Figure 1a). Late growing season warming was evident in all years with the 5 yr mean fall Ta being 2°C (Figure 2a; Table 2) warmer than the 30‐yr mean. Fall was the only season where mean seasonal Ta was higher in all years compared to the 30‐yr mean.

**Table 2:** Seasonal Values of Carbon Fluxes and Meteorological Variables

	Season	2012	2013	2014	2015	2016	5 yr mean
Total NEP (g C m^−2^)	Spring	−17	−23	−58	−32	−71	−40
	Summer	379	287	448	265	373	350
	Fall	8	−50	−16	−65	−30	−31
	Winter	−50	−60	−61	−66	−98	−67
	Annual	292	156	305	90	185	206
Total GEP (g C m^−2^)	Spring	179	153	100	159	117	142
	Summer	787	936	980	907	945	911
	Fall	232	280	303	281	357	291
	Winter	—	—	—	—	—	—
	Annual	1198	1369	1382	1347	1420	1343
Total RE (g C m^−2^)	Spring	214	182	173	205	204	196
	Summer	408	648	531	638	570	559
	Fall	234	339	323	351	389	327
	Winter	60	90	78	74	108	82
	Annual	954	1250	1110	1283	1260	1171
Total *P* (mm)	Spring	115	214	233	190	231	197 (236)
	Summer	159	267	244	206	144	204 (255)
	Fall	311	256	373	218	311	294 (266)
	Winter	143	206	188	104	189	166 (218)
	Annual	801	954	991	750	908	881 (997)
Mean Ta (°C)	Spring	11.7	7.50	5.66	7.20	7.89	7.99 (6.87)
	Summer	22.9	20.3	20.1	19.9	22.0	21.0 (20.0)
	Fall	11.4	10.8	10.7	13.1	13.6	11.9 (9.90)
	Winter	0.27	−0.14	−5.56	−5.40	0.49	−2.07 (−3.85)
	Annual	11.84	9.23	8.02	9.15	10.56	9.76 (8.0)
Mean PAR (μmol m^−2^)	Spring	369	324	343	348	333	343
	Summer	467	428	449	444	480	454
	Fall	201	201	201	225	225	211
	Winter	124	163	146	102	102	127
	Annual	291	279	285	280	285	284
Mean VPD (kPa)	Spring	0.85	0.54	0.43	0.50	0.54	0.57
	Summer	1.33	0.75	0.79	0.77	1.01	0.93
	Fall	0.61	0.45	0.47	0.55	0.52	0.52
	Winter	0.23	0.41	0.26	0.29	0.38	0.31
	Annual	0.75	0.54	0.49	0.53	0.61	0.58

*Note*. Winter values for each year used December values from the previous year. Values of Ta and *P* in parentheses are the 30‐yr (1981 to 2010) mean values from the ECCC Delhi station.

Annual *P* in 2012 (801 mm) was 50 mm below the 30‐yr mean *P* value of 997 mm (ranging from 852–1,142 mm; Figure 2a and Table 2). *P* was strikingly absent from May through July in 2012. Following a wet March and April 2016 experienced the lowest summer *P* (144 mm) of the 5 yr, although similar to the dry summer of 2012 (159 mm). On average, 64 days yr^−1^ saw *P* greater than 5 mm (0.2 in) over the 30‐yr (1981 to 2010) period, while extreme *P* events in excess of 25 mm day^−1^ occurred 7 days yr^−1^. During the study period (2012 to 2016), extreme daily *P* events (>25 mm day^−1^) occurred on 8 days in 2013, 9 days in 2014, and 4 days in 2016. *P* events occurred quite frequently in 2016, with 53 days receiving *P* greater than 5 mm.

**Figure 3:**
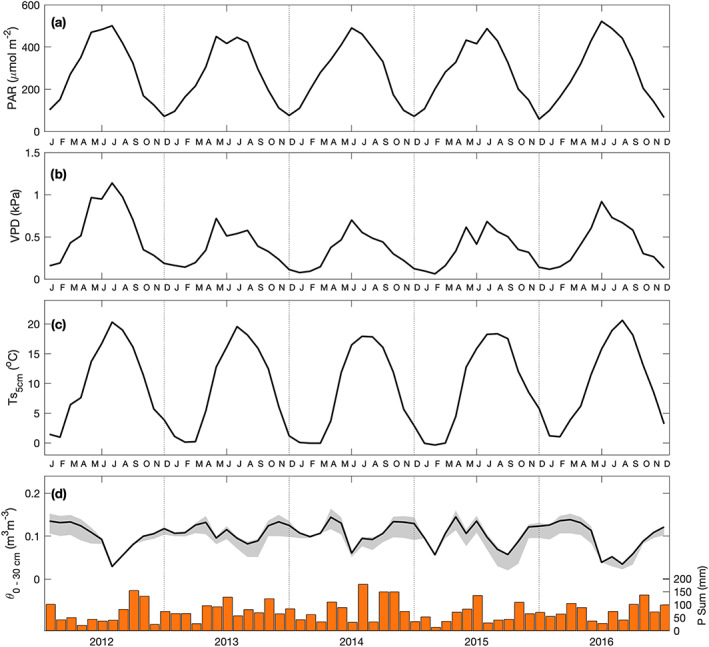
Monthly mean values of (a) photosynthetically active radiation (PAR), (b) vapor pressure deficit (VPD), (c) 5 cm soil temperature (Ts), and (d) mean volumetric water content (*θ*) between 0 and 30 cm depths (left axis) and daily total *P* values (right axis) from 2012 to 2016. Gray shading in (d) illustrates the range (5–50 cm) of *θ* within the 0–30 cm soil column.

Monthly mean downward PAR followed similar year‐to‐year patterns (Figure 3a). Mean June PAR yielded a maximum in 2016 (522 μmol m^−2^ month^−1^), but a clear decrease in June PAR was observed in 2013 (417 μmol m^−2^ month^−1^) and 2015 (416 μmol m^−2^ month^−1^). Large declines in PAR were often associated with increased cloudiness or *P* events, as seen in 2013 and 2015 (Figure 3d). High PAR was associated with high Ta and VPD, observed in 2012 and 2016 (Figure 1a).

Temporal variability in soil temperature (Ts) closely followed the temporal variability of Ta (*R* ^2^ = 0.88; Figures 1a and 3c). While maximum Ts_5cm_ occurred during the summer months, the timing of the maximum Ts varied year to year, often occuring during prolonged periods of decreased *P* or volumetric soil water content (*θ*). The mean monthly *θ* (Figure 3d) ranged from a minimum of 0.030 m^3^ m^−3^ in July of 2012, to a maximum of 0.145 m^3^ m^−3^ in April of 2014. The 5 yr mean *θ*_0‐30cm_ was 0.104 m^3^ m^−3^. Deep declines in *θ* were observed during summer months characterized by high Ta and little to no *P* (i.e., 2012, 2014, and 2016). In 2012, a significant decrease in *θ*_0‐30cm_ occurred over a 63 day period (June–July), before substantial rain (>25 mm) was received. Similarly in 2016, a near 3 month (May–July) decrease in *θ*_0‐30cm_ was the second lowest monthly *θ* recorded (0.035 m^3^ m^−3^). In all years, *θ* was the highest in the spring (after snowmelt), before declining with the onset of photosynthetic activity at the start of the growing season. The depletion of *θ* typically lasted the majority of the summer, before being replenished near the end of the growing season in fall due to high *P* and decreased atmospheric and physiological water demand. During winter, mean *θ*_0‐30cm_ values typically ranged between 0.1 and 0.15 m^3^ m^−3^ (Figure 3d).

### 3.2 Carbon Fluxes

**Figure 4:**
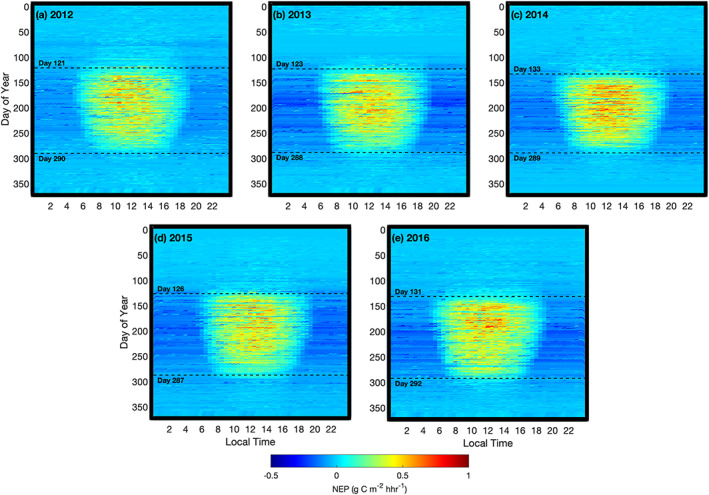
(a–e) Half‐hourly net ecosystem productivity (NEP) values plotted for each half hour of the day (local time, *x* axis) and day of year (*y* axis) from 2012 to 2016. The timing of leaf‐out (spring) and leaf fall (fall) determined from PhenoCam transition dates are labeled by dashed lines. Missing NEP data were gap filled following methods outlined in section 2.5. Half‐hourly GEP and RE values are shown in supporting information Figures S1 and S2, respectively.

Half‐hourly flux data indicating the diurnal and annual variations in NEP are shown in Figure 4. Cool, blue colors symbolize a carbon source or release (negative NEP) and warmer yellow, orange, and red colors represent carbon uptake by the forest (positive NEP). Typical midday NEP values were near 0.5 g C m^−2^ half‐hour (hhr)^−1^, with maximum NEP values reaching nearly 1 g C m^−2^ hhr^−1^ (Figure 4). The source‐sink transition of CO_2_ fluxes, or the start of the growing season, occurred in early May (day of year (DOY) 127; 7 May) on average with maximum NEP values observed in early June. The growing season continued until mid‐October (DOY 292; 19 October). Due to the higher laititude, positive NEP occurred for as long as 12 to 13 hr over a large portion of the growing season. The start and end of the growing season varied from year to year by up to 12 days. During the leafless periods (winter) or nights, the site was a source of carbon with negative NEP values observed.

**Figure 5:**
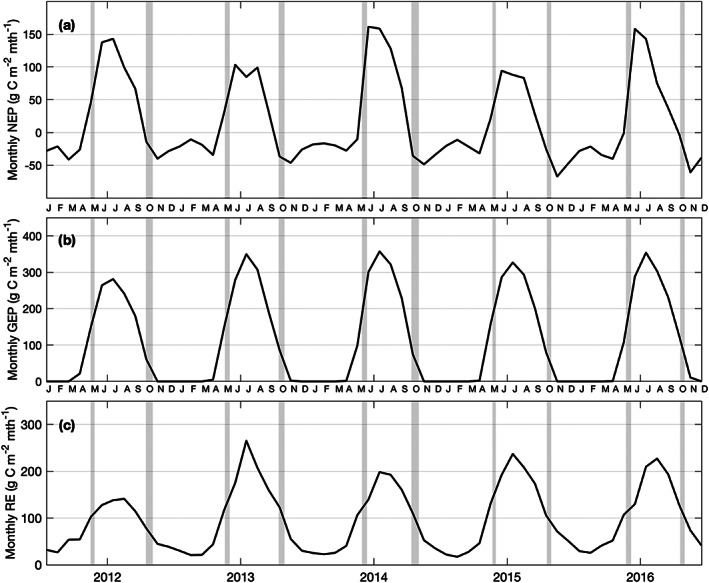
Monthly total (a) net ecosystem productivity (NEP), (b) gross ecosystem productivity (GEP), and (c) ecosystem respiration (RE) from 2012 to 2016. Gray shading shows the 10–50% range of transition dates from PhenoCam greenness chromatic coordinate (GCC) measurements.

**Figure 6:**
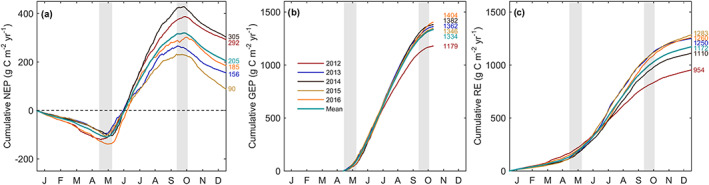
(a) Cumulative values of daily (a) NEP, (b) GEP from DOY 119 to 300 (encompasses all years), and (c) RE from 2012 to 2016. Gray shading (same as Figure 5) depicts the range of growing season transition dates for the 5 yr of data. Annual total values are also shown.

Low daily NEP values were recorded in the growing season of both 2013 and 2015 (Figures 4b and 4d). In 2014, the growing season began much later than other years (DOY 133; 13 May), however, despite the late start of the growing season, the year had the largest carbon uptake or highest annual NEP value of all years (Figure 4c). In 2016, the extended length of the growing season allowed positive NEP values to be recorded until the end of October (Figure 4e). Monthly mean and cumulative C fluxes (GEP, RE, and NEP) are shown in Figures 5 and 6, respectively, while seasonal and annual value are shown in Table 2. At the beginning of each year, the forest was a C source (negative NEP), due to the absence of leaves on trees and no photosynthesis. Colder temperatures caused relatively low RE. Following leaf‐out, increasing PAR combined with warmer Ta, helped the photosynthetic flux (GEP) to increase and outweigh respiratory fluxes (RE). Peak daily NEP (maximum C uptake) was reached in June each year (Figure 5a). Opposing environmental controls such as high PAR and high Ta and VPD during the summer often limited the overall GEP. The year with the highest VPD (2012) resulted in the lowest GEP.

The maximum values of monthly GEP and RE were observed in the middle of summer (in July) during all years, when Ta and corresponding Ts values were highest (Figure 1a and Figure 3c). During the second half of each summer, NEP steadily declined until leaf senescence occurred and the growing season ended. At this point, NEP became negative and the site was a C *source*. The highest annual NEP was observed in 2014 (305 g C m^−2^ yr^−1^), followed closely by 2012 (292 g C m^−2^ yr^−1^). The remaining 3 yr had annual NEP values less than 200 g C m^−2^ yr^−1^, with 2015 being the lowest at 90 g C m^−2^ yr^−1^ (Figure 6a). The spring of 2016 experienced a delayed start to the growing season similar similar to 2014, which had the highest annual NEP, however the annual C sink strength of 2016 (185 g C m^−2^ yr^−1^) was just over half of 2014 (Figure 4). NEP in 2016 fell short of the warmest (2012) and wettest (2014) years, due to warm summer and fall Ta.

### 3.3 Environmental Controls on Carbon Fluxes

A multivariate linear regression analysis was performed using non‐gap‐filled C fluxes and explanatory meteorological and soil variables (i.e., gap‐filling methods) at half‐hourly and daily time scales, throughout the growing season, to quantify the relative explanatory power of the individual environmental variables (PAR, VPD, Ts_5cm_, and *θ*_0‐30cm_) in explaining the temporal variability of C fluxes. The *R* ^2^ of partial models were also compared to the full model *R* ^2^ (Table 3). GEP, RE, and NEP residuals had normal distributions at our site. The models explanatory power was highest for RE at both half‐hourly and daily times scales (*R* ^2^ ~ 0.8). NEP was the only flux to have a higher *R* ^2^ for the daily model when compared to the half‐hourly model (Table 3). Ts_5cm_ and PAR were the main explanatory variables for half‐hourly GEP, accounting for 43% and 41% of the total variability explained, respectively. Ts_5cm_ was the primary variable that explained most of the variability in RE at both half‐hourly and daily time scales. Ts_5cm_ was able to best predict RE because it captured both soil processes and phenological effects over the growing season due to its close correlation with Ta in this deciduous forest. Ts_5cm_ explained more variability in NEP at the daily time scale, however, it was the secondary explanatory variable on the half‐hourly time scale, behind PAR. *θ*_0‐30cm_ acted to increase RE, though it failed to explain GEP and NEP variability. Additionally, VPD was found to have a minor impact on NEP and GEP. Overall, during the 5 yr of the study, temperature was the dominant environmental control, where Ts_5cm_ explained 95%, 68%, and 32% of the variability in daily RE, GEP, and NEP, respectively, while PAR explained 9% and 19% of the variability in daily GEP and NEP, respectively. *θ*_0‐30cm_ explained 13% and 9% of the variability in RE at both scales, but was not a major explanatory variable for GEP and NEP.

**Table 3:** Relative Contribution of Explanatory Variables

	Daily			Half‐hourly		
	NEP	GEP	RE	NEP	GEP	RE
PAR (% *R* ^2^)	18.5	8.89	NS	58.5	41.3	0.17
VPD (% *R* ^2^)	NS	1.93	3.69	11.1	11.0	1.83
Ts _5cm_ (% *R* ^2^)	32.2	68.3	95.3	24.3	43.2	97.1
*θ*_0‐30cm_ (% *R* ^2^)	2.20	0.49	9.32	0.58	NS	12.9
Model *R* ^2^	0.32	0.70	0.82	0.47	0.61	0.81
*F* statistic (*α* < 0.001)	94	462	886	4.03E3	7.02E3	1.93E4

*Note*. Contribution of explanatory variables to the multivariate models of daytime, growing season net ecosystem productivity (NEP), gross ecosystem productivity (GEP), and ecosystem respiration (RE) and model performance. Analysis was performed at daily and half‐hourly time scales, using non‐gap‐filled data. NS indicates that a variable was not significant in the model.

**Figure 7:**
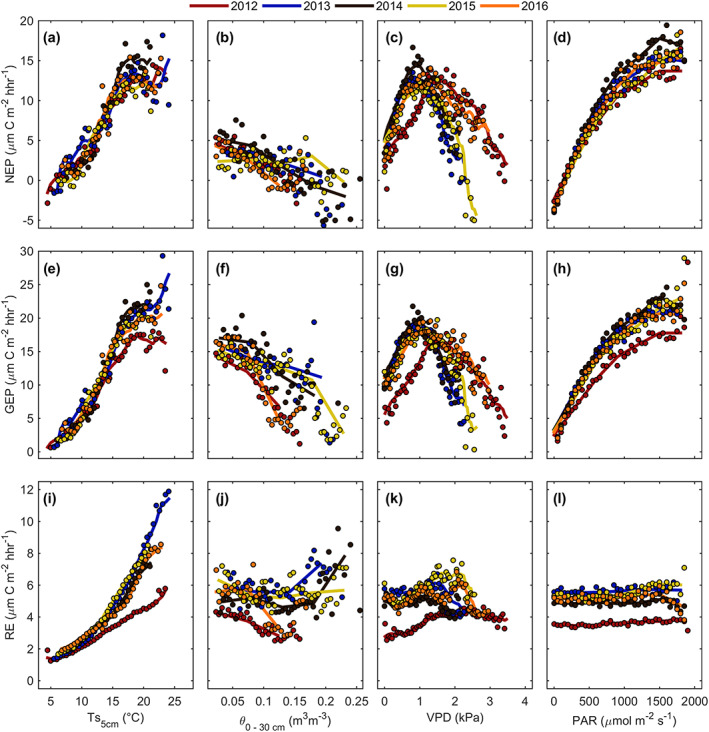
(a) Non‐gap‐filled growing season net ecosystem productivity (NEP) and bin‐averaged 5 cm soil temperature (Ts_5cm_, bin of 0.5°C), (b) bin‐averaged 0–30 cm volumetric water content (*θ*_0‐30cm_, bin of 0.005 m^3^ m^−3^), (c) bin‐averaged vapor pressure deficit (VPD, bin of 0.05 kPa), and bin‐averaged photosynthetically active radiation (PAR, bin of 50 μmol m^−2^ s^−1^. (e–h) Same analysis for non‐gap‐filled gross ecosystem productivity (GEP) and (i–l) ecosystem respiration (RE). Solid lines are annual moving average fits.

Bin‐averaged daytime, half‐hourly values of NEP (non‐gap‐filled), GEP, and RE over the growing season, plotted against the key meteorological variables (e.g., PAR, VPD, Ts_5cm,_ and *θ*_0‐30cm_) are shown in Figure 7. All years experienced a steady rise in NEP (Figure 7a) and GEP (Figure 7e) following an increase in Ts_5cm_ until it reached 15–20°C. Beyond this range of Ts, both NEP and GEP began to plateau or even decrease with increasing Ts. An exponential increase in RE (Figure 7i) with increasing Ts_5cm_ was seen in all years. During 2014, the year with the highest annual NEP, maximum half‐hourly NEP reached nealy 17 μmol C m^−2^ hhr^−1^ when Ts_5cm_ was 20°C, which was considerably higher than other years. Even within the ideal range of Ts (15–20°C), NEP and GEP were limited due to stomatal controls during high VPD and soil water limitiation periods (Table 3). As Ts_5cm_ increased, *θ*_0‐30cm_ decreased, illustrating how Ts and *θ* were highly correlated at our site. The years with the highest GEP and NEP were also the wettest, especially during the summer (2013 and 2014). While all years saw a decrease in NEP (Figure 7b) and GEP (Figure 7f) with increasing *θ*_0‐30cm_, GEP however was sustained at higher *θ*_0‐30cm_ values in 2013 and 2014. The hot and dry years (2012 and 2016) experienced a large decrease in GEP at moderate *θ*_0‐30cm_, as limitations in water availability likely impacted stomatal function.

Comparisons of bin‐averaged VPD with GEP and NEP illustrated that C fluxes were typically low at very low VPD (<0.5 kPa), before increasing with increasing VPD values and then declining again when VPD exceeded 1.5 kPa (Figures 7c and 7g). This trajectory displayed high rates of photosynthesis and net C uptake during sufficiently moist atmospheric conditions and a rapid decrease in photosynthesis and net C uptake with increasingly dry atmospheric conditions. Maximum NEP (16 μmol C m^−2^ hhr^−1^) and GEP (21 μmol C m^−2^ hhr^−1^) values were observed over a VPD range of 0.7 to 1.2 kPa (Figures 7c and 7g). In 2012, high Ts and very high VPD (Figure 3b) over an extended time period led to a considerable deviation from other years, where maximum photosynthetic fluxes were observed as VPD values approached 1.4 to 1.5 kPa. In all years, RE saw nearly constant half‐hourly fluxes for all ranges of VPD (Figure 7k). Lastly, bin‐averaged PAR and NEP (Figure 7d), GEP (Figure 7h), and RE (Figure 7l) were examined. GEP and NEP values rapidly increased with increasing PAR, however, the rate of their increased started to plateau when PAR exceeded roughly 800 μmol m^−2^ s^−1^, before a slight decline at very high PAR values (Figures 7h and 7d). All years exhibited similar patterns of increasing GEP and NEP when compared to PAR, except for 2012, which experienced a heat wave and drought during the early growing season.

### 3.4 Regional Analysis

Annual values of C fluxes measured at our site were compared to fluxes from four other eastern North American temperate deciduous forests from 2012 to 2016. A fifth site, Silas Little, New Jersey, USA (US‐Slt), was also considered. However, a major insect defoliation in 2007 resulted in the site being a large C source for 2 yr after the disturbance. During the postdisurbance years (2009–2016), mean annual NEP (37 ± 18 g C m^−2^ yr^−1^) was only 22% of the NEP observed during the predisturbance years (2004–2006) (Clark et al., 2018). Therefore, this site was excluded from the comparison. Annual values of GEP, RE, and NEP, along with site characteristics and meteorological variables are given in Table 4. The ages of the forests ranged from 70 to 130 yr, with the youngest being Harvard Forest in Petersham, Massachusetts (Urbanski et al., 2007) and the oldest being the Bartlett Experimental Forest in Durham, New Hampshire (Lee et al., 2018). For all forests, the mean annual Ta and *P* estimated from available site data ranged from 5.2 to 14.5°C and 560 to 2,384 mm, respectively. On an annual basis, the forests were sinks of C with annual NEP from 69 to 459 g C m^−2^ yr^−1^, GEP from 1,180 to 1,684 g C m^−2^ yr^−1^, and RE from 954 to 1,406 g C m^−2^ yr^−1^. The 5 yr mean values of NEP (206 g C m^−2^ yr^−1^), GEP (1,343 g C m^−2^ yr^−1^), and RE (1,171 g C m^−2^ yr^−1^) at our site fell just slightly below the mean values for all sites, but within the range of standard deviations for all sites included in the regional analysis (Table 4).

**Table 4:** Summary of Regional Analysis Site Characteristics

Site	Location	Species	Ta (°C)	*P* (mm)	Year	Age	NEP (g C m^−2^)	GEP (g C m^−2^)	RE (g C m^−2^)	Site reference
Coweeta (US‐Cwt)	35.0592; −83.4275	Tulip, poplar, white oak, black birch, red maple	13	1,495	2012	81	194	1,563	1,369	Novick et al. (2013) Oishi et al. (2018)
					2013	82	364	1,602	1,238	
					2014	83	315	1,552	1,237	
					2015	84	147	1,463	1,316	
Harvard Forest (US‐Ha1)	42.5369; −72.1725	Red oak, red maple, eastern hemlock	6.6	1071	2012	74	339	1,684	1,345	Urbanski et al. (2007) Munger and Wofsy (2017)
					2013	75	218	1,495	1,277	
					2014	76	459	1,557	1,098	
					2015	77	194	1,600	1,406	
Turkey Point Deciduous (CA‐TPD)	42.6353; −80.5577	White oak, sugar maple, red maple, American beech	8	1,036	2012	90^a^	292	1,198	954	This Study
					2013	91^a^	156	1,369	1,250	
					2014	92^a^	305	1,382	1,110	
					2015	93^a^	90	1,347	1,283	
					2016	94^a^	185	1,420	1,260	
Bartlett (US‐Bar)	44.0639; −71.2874	Red maple, sugar maple, paper birch, American beech	6.6	1,300	2012	110^a^	114	1,291	1,177	Lee et al. (2018)
					2013	111^a^	120	1,309	1,189	
					2014	112^a^	102	1,314	1,200	
					2015	113^a^	110	1,268	1,166	
					2016	114^a^	69	1,378	1,269	
University of Michigan Biological Station (US‐UMB)	45.5598, −84.7138	Red oak, sugar maple, red maple, bigtooth aspen	5.5	817	2012	89	331	1,309	978	Gough et al. (2013)
					2013	90	214	1,180	966	
					2014	91	172	1,299	1,127	
					2015	92	229	1,315	1,081	
					2016	93	182	1,318	1,131	
Mean			7.94	1,144		91.6	213 ± 102	1,401 ± 138	1,192 ± 124	

*Note*. List of site characteristics, mean annual Ta, total annual *P*, and annual carbon fluxes (NEP, GEP, and RE) of eastern North American deciduous forests, from 2012 to 2016.

^a^ Mean forest age; CA‐TPD: 70–110 yr; US‐Bar: 90–130 yr.

**Figure 8:**
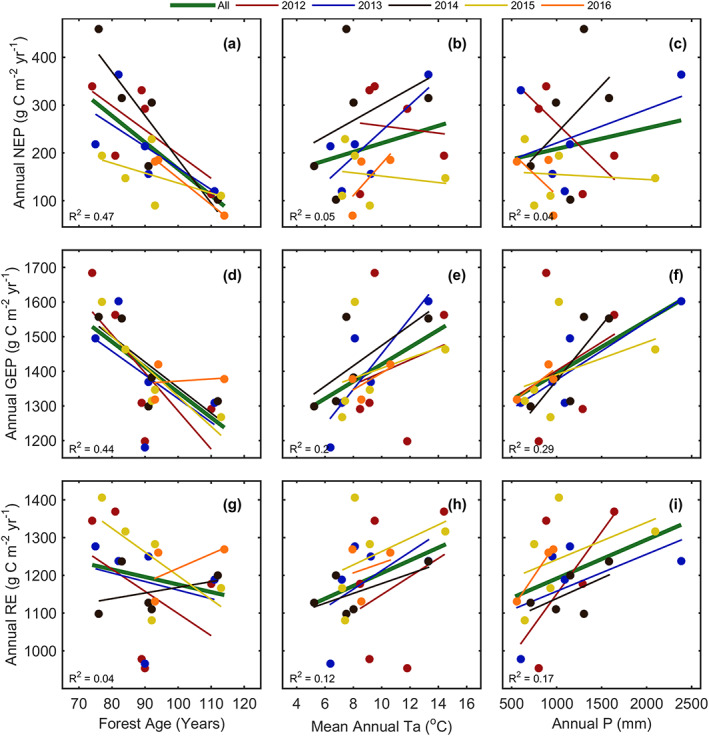
Relationships between observed (a–c) NEP, (d–f) GEP, and (g–i) RE values from 2012 to 2016 and site characteristics of sites included in the regional analysis, including forest age, mean annual air temperature (Ta), and total annual precipitation (*P*). The fit of each year is color coded, while the *R* ^2^ values over the 5 yr of data are shown as a dark green line.

GEP, RE, and NEP values from each site included in this comparison were plotted against individual site characteristics, including forest age, mean annual Ta, and total annual *P* as shown in Figure 8. No signficant relationships between annual fluxes and latitude were found. This was illustrated by the similarities in annual GEP, RE, and NEP values between the Coweeta forest (35°N) and Harvard forest (42.5°N), even though site Ta and *P* varied considerably (Table 4). Forest age was shown to be negatively correlated to NEP and GEP (Figures 8a, 8d, and 8g). The 5 yr mean fit found that an increase of 10 yr in a forest's age caused a decrease in annual NEP by 56 g C m^−2^ yr^−1^, with a similar decrease experienced in GEP values (73 g C m^−2^ yr^−1^). Mean annual Ta (Figure 8b) and total *P* (Figure 8c) had little influence on annual NEP, largely varying between individual years. Interestingly, the driest and warmest years (2012, 2015, and 2016) led to decreases in NEP. Overall, in all years, annual GEP increased with increasing mean annual Ta (Figure 8e) and total annual *P* (Figure 8f). The 5 yr mean fit found that an increase of 2°C in Ta and 200 mm in *P* would result in a 50 and 30 g C m^−2^ yr^−1^ increase in annual GEP and NEP, respectively. No significant relationships were found between RE and forest age (Figure 8g) or mean annual Ta (Figure 8h), although annual RE increased with increasing annual *P* (Figure 8i).

## 4 Discussion

### 4.1 Carbon Fluxes and Environmental Controls

C budgets of temperate deciduous forests are unique due to their distinct seasonal patterns of C gain and loss over the course of a year, which is driven largely by leaf phenology and seasonal meteorological conditions (Greco & Baldocchi, 1996). At our site, photosynthesis (GEP) was initiated between mid‐April and early May (DOY 105 to 121) and ended by the end of October to mid‐November (DOY 300 to 314), with the 5 yr mean start of the growing season occurring on 7 May (DOY 127) and ending on 16 October (DOY 289). In winter, the site was a small but consistent C source (i.e., negative NEP) due to respiratory fluxes (RE) and no photosynthesis (Table 2). During this time, daily NEP (RE = −NEP) values averaged between negative 1–2 g C m^−2^ day^−1^. Similar winter NEP values have been observed at other deciduous forests (Bolstad et al., 2004; Curtis et al., 2002). Following the winter and into early spring until leaf‐out, the site continued to become a stronger C source following increasing Ta and hence Ts (Figure 6a). Following leaf‐out, however, NEP rapidly increased and the site became a C sink, achieving maximum NEP values by early June. At our site, the spring of 2012 (Table 2) had the highest spring Ta (11.7°C), which was ~3°C higher than any other year. This led 2012 to have the earliest growing season start and highest spring NEP (17 g C m^−2^), similar to the observations made by Richardson et al. (2010). Ultimately, this early start of the 2012 growing season aided the year in having the second highest annual NEP.

The key period which determined the overall annual NEP at our site was late spring to midsummer (late May to the middle of July). What happened in this period would determine the overall strength of the annual C sink at our site. For example, in 2013 and 2015, the two years with the lowest annual NEP values, the growing season began at similar times and C uptake rates steadily increased until mid‐July (Figure 6a), before declining and producing much smaller annual C uptake as compared to other years. These two years had the lowest spring *P* values at the start of the growing season and then the lowest PAR values in the summer (Table 2 and Figure 3a). In 2015, soil water deficits (*θ*_0‐30cm_) approached a minimum near the end of July; while in 2013, deficits were not observed due to a few large *P* events in the early summer, leading NEP to be slightly higher in 2013 as compared to 2015 (Figure 3d). Consequently, 2015 had the lowest summer and annual NEP values, followed by 2013. In 2012 and 2014, the two years with the highest annual NEP on record, the start of the growing season varied by 3 weeks, but both years experienced similar C uptake up until July (Figure 6a). The year 2012 experienced very warm temperatures from the beginning of the year which continued well into the summer (Figure 1a and Figure 2a). A drought and lull in *P* (Figure 1b) also occurred from May to July in 2012, but was replenished by ample *P* in late July. While the NEP of both years started at similar rates, the heat stress in combination with a drought caused much lower RE values as compared to GEP in 2012 (Figures 5 and 6). This disparity in the response of GEP and RE caused higher NEP values leading 2012 to become the second most productive year of the study (Figure 6a). Relatedly, 2014 and 2016 had similar growing season starts but experienced a large deviation in C uptake following a decrease in *θ*_0‐30cm_ in the spring as observed in 2012. This showed that soil water status and meteorological conditions (temperature and radiation) during spring and midsummer were major factors to determine the annual NEP values in our forest. More importantly, the timing, duration and severity of these meteorological conditions shaped the overall C dynamics of the forest. Similar observations have been made by Wu et al. (2012) and Zscheischler et al. (2016) at temperate forest sites.

An analysis of the major environmental controls (e.g., PAR, Ta, Ts_5cm_, VPD, and *θ*_0‐30cm_) on the carbon fluxes at our site further showed that the variability in NEP was primarily driven by changes in RE, rather GEP (Table 3 and Figures 6b, 6c, and 7). Most years had very similar GEP values, except 2012, when seasonal and annual RE values revealed large variations. During the summer, high Ta (>20°C) and high VPD (>1 kPa) coincided with high Ts_5cm_ and increased soil water deficits. Overall, higher temperatures and dry conditions inhibited RE much more than GEP, leading to higher NEP (Figures 5 and 6). In the spring and early summer soil water status (*θ*) played an important role in shaping the overall dynamics of RE, GEP, and hence NEP. In the summers of 2012 and 2016, monthly *θ*_0‐30cm_ dropped well below the mean values observed at our site (i.e 0.104 m^3^ m^−3^) and reached as low as 0.05 m^3^ m^−3^ (Figure 3d). Low soil moisture and water stress caused both GEP and RE to decline when regressed against *θ*_0‐30cm_ (Figures 7f and 7j), however, 2012 experienced a much larger decrease in RE than in 2016. These results showed that temperaure (e.g., Ts_5cm_) was the dominant control on RE and therefore NEP at our site (Table 3). Higher temperatures significantly reduced RE and this reduction increased when warmer temperatures occurred at the same time as drought conditions, as experienced in 2012. An earlier study by Froelich et al. (2015) in a mixed wood forest north of our site in the temperate–boreal ecotone near Borden, Ontario (44°19′N, 79°56′W), suggested that PAR and soil temperature were main driving factors for the interannual variability in NEP, particularly during the summer (June to August) period. They also found that longer growing seasons increased NEP at this site. Similarly, Richardson et al. (2009) found warm spring Ta helped to enhance GEP through the lengthening of the growing season, however, increased heat or water‐stressed conditions and high summer Ta reduced GEP, while warmer soils enhanced RE in deciduous forests (Gallinat et al., 2015). Our study also found that high Ta in the fall may lead to a lengthening of the growing season through October and early November and a subsequent reduction in C uptake through enhanced RE. The resulting late season C losses prior to leaf senescence often outweigh the gains from prolonged photosynthesis due to longer growing seasons (Froelich et al., 2015; Piao et al., 2008; Richardson et al., 2010; Schimel, 1995). In all years, even during heat and water‐stressed conditions, our site remained an annual C sink. However, with more extreme Ta and variability in *P* expected in the future, the extension of an already hot and dry growing season could further promote carbon loss, affecting the annual sink‐source status of many temperate deciduous forests.

### 4.2 Regional Analysis

Without human disturbances, deciduous forests would be the dominant forest type in the temperate climate region across eastern North America (Curtis et al., 2002). These forests are estimated to sequester an average of 1.0 to 2.4 Mg C ha^−1^ yr^−1^ or 100 to 240 g C m^−2^ yr^−1^ (Birdsey, 1992). Our study compared the annual C fluxes and their driving factors in North American temperate deciduous forests. For the forests analyzed in our study, the mean annual NEP was 213 ± 102 g C m^−2^ yr^−1^ (Table 4). The lowest NEP values were recorded in northern latitude sites in the United States (Michigan and New Hampshire) and southern Ontario Canada. Although not included in our regional analysis, the aforementioned mixed wood Borden forest (Froelich et al., 2015), composed of red maple, white pine, aspen and ash trees, had an average NEP of 177 g C m^−2^ yr^−1^ over 17 yr (1996–2013) of measurements, with the forest being a weak C source for two years (1996 and 2001) during the measurement period. Higher NEP was measured further south at the Walker Branch deciduous forest in Oak Ridge, Tennessee (35°N), averaging 574 g C m^−2^ yr^−1^ during 5 yr (1995–1999) of measurements (Wilson & Baldocchi*,* 2000). While the most southern forest in our analysis (e.g., Coweeta) had high annual NEP, we found no relationship between latitude and annual NEP.

The University of Michigan Biological Station (UMBS) forest in Northern Michigan lies within the transition zone between mixed hardwood forests and boreal forests and has similar stand age, soil characteristics, tree species and latitude as our site (Gough et al., 2013). At their site the NEP ranged from 70 to 170 g C m^−2^ yr^−1^ over a 3‐yr period from 1998 to 2001 (Curtis et al., 2002; Schmid et al., 2003). However, in recent years (2012–2016), NEP at their site ranged from 172 to 331 g C m^−2^ yr^−1^ (Table 4). At our site, NEP ranged from 90 to 305 C m^−2^ yr^−1^, which was well within the range of similar forests. These similar sites yielded a negative correlation (*R* ^2^ ~ 0.5) between forest age and annual NEP, though other site variables could not explain NEP. Eastern deciduous forests are typically a moderate C sink, but their C sink capacity varies due to meteorological conditions. The studies at the Oak Openings deciduous forest in northwestern Ohio suggested that seasonal GEP was determined by Ta, *θ*, and VPD, while year‐to‐year differences were explained by changes in LAI. Additionally, RE was most sensitive to Ta and *θ*, while the *θ* effect on RE may have been facilitated by GEP (Noormets et al., 2008; Xie et al., 2014). The main meteorological factors driving variability in the C fluxes at our site were Ts (driven by Ta) followed by PAR. No significant contribution from *θ* was found for NEP, but soil water stress was shown to reduce RE, which contributed to offset its impact on NEP. Our study and the regional analysis highlight the importance of both seasonal environmental and phenological influences on C exchanges in temperate deciduous forests across eastern North America, while helping to explore how these forests may respond to future climate change.

## 5 Conclusions

Seasonal and annual dynamics of C fluxes were studied for 5 yr (2012 to 2016) at a mature temperate deciduous forest in the Great Lakes region of southern Ontario, Canada. On average, the forest was an annual C sink of 206 ± 92 g C m^−2^ yr^−1^, which was similar to the mean annual NEP values observed at other deciduous forests in eastern North America. Meteorological conditions during the spring and early summer greatly impacted annual NEP values. PAR was the major explanatory variable for half‐hourly NEP values. However, temperature (Ta and Ts) and water availability (*P* and *θ*) during the summer period helped to define the strength of the annual C uptake. Temperature (e.g., Ts_5cm_) was the dominant control on GEP and RE and hence a key controlling factor of interannual NEP at our site. Higher temperatures significantly reduced RE, and this reduction was much more pronounced when these warmer temperatures occurred simultaneously with drought conditions, as experienced in 2012. Although heat and drought reduced GEP, the reduction in RE was found to be higher, causing NEP (i.e., NEP = GEP − RE) to increase. This was observed in 2012 which was the second most productive year in terms of NEP despite experiencing hot temperatures and drought conditions in the earlier parts of the year. These results illustrate that the net C sequestration capabilities of our oak‐dominated forest were resilient to environmental stresses, and despite the significant environmental variability and extreme weather events that occurred over the 5 yr, the forest was still a strong C sink. It will be important to observe how the C uptake of this forest may respond to future climatic changes which are expected to become more severe with the passage of time.

## Conflict of Interest

The authors declare no competing interests.

## Supporting information

Supporting Information S1

Figure S1

Figure S2

## Data Availability

Data used in this study can be found and accessed online (at https://ameriflux.lbl.gov/sites/siteinfo/CA-TPD).
