# Supplementary material for: The Impact of Seasonal and Annual Climate Variations on the Carbon Uptake Capacity of a Deciduous Forest Within the Great Lakes Region of Canada
Source: J Geophys Res Biogeosci. 2020 Sep 18;125(9):e2019JG005389. doi: 10.1029/2019JG005389 (PMC7540005; doi:10.1029/2019JG005389)
Supplement: Supplementary file 1 — Supporting Information S1 [file JGRG-125-e2019JG005389-s001.pdf]

*Journal of Geophysical Research: Biogeosciences*

Supporting Information for:

# The impact of seasonal and annual climate variations on the carbon-sink capacity of a deciduous forest within the Great Lakes Region of Canada

**Eric R. Beamesderfer[1], M. Altaf Arain[1*], Myroslava Khomik[1, 2], and Jason J. Brodeur[1]**

[1]School of Geography and Earth Sciences and McMaster Centre for Climate Change, McMaster University, Hamilton, Ontario, Canada

[2]Geography and Environmental Management, University of Waterloo, Waterloo, Ontario, Canada

The supporting information includes: seven pages, two figures, and two tables

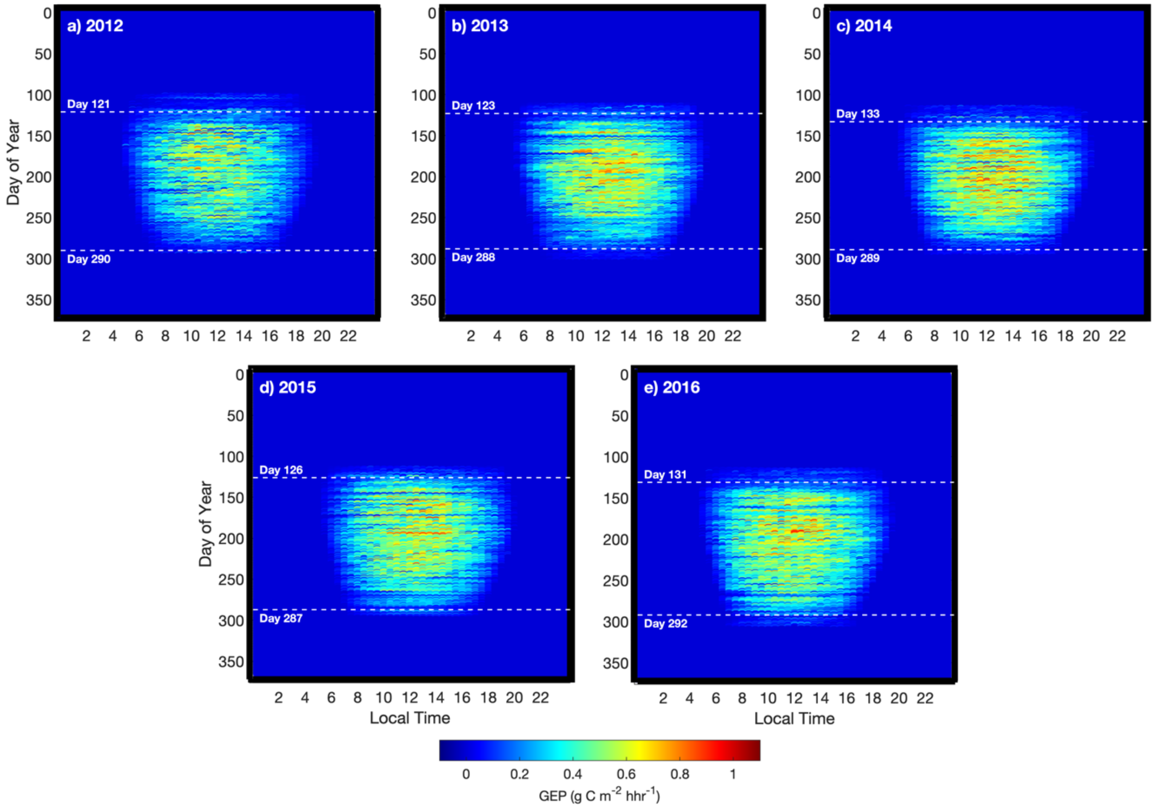


**Figure S1.** (a – e) Gap-filled half-hourly Gross Ecosystem Productivity (GEP) plotted for each half hour of the day (local time, x-axis) and day of year (y-axis) from 2012 to 2016. Timing of leaf-out and leaf-fall determined by PhenoCam transition dates are labeled by dashed lines with corresponding day of year (DOY).

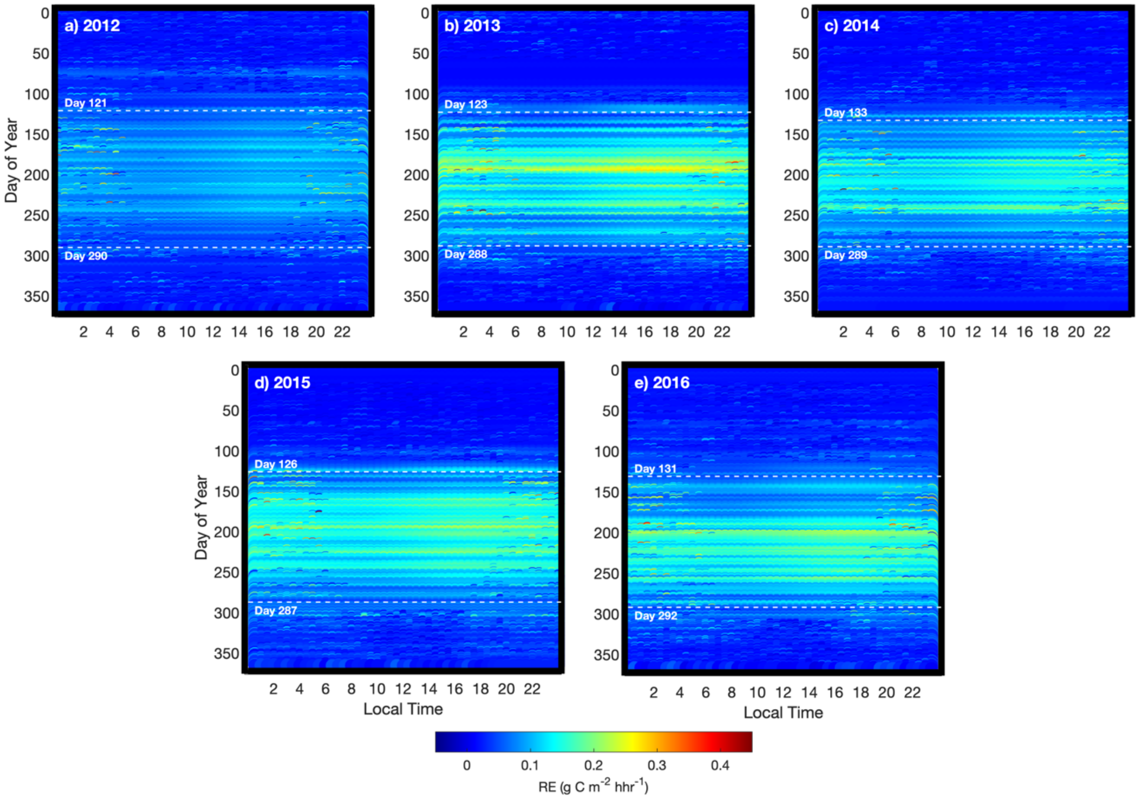


**Figure S2**. (a – e) Gap-filled half-hourly Ecosystem Respiration (RE) plotted for each half hour of the day (local time, x-axis) and day of year (y-axis) from 2012 to 2016. The dates of spring leaf-out and autumn leaf-fall determined by PhenoCam transition dates are labeled by dashed lines with corresponding day of year (DOY).

| | Model | $R^2$ | AIC | BIC |
|---|---|---|---|---|
| **a) NEP (daily)** | VPD | 0.0625 | 3631 | 3641 |
| | PAR | 0.1219 | 3579 | 3588 |
| | $\theta_{0\text{-}30cm}$ | 0.1336 | 3568 | 3578 |
| | PAR + VPD | 0.1403 | 3564 | 3578 |
| | VPD + $\theta_{0\text{-}30cm}$ | 0.1439 | 3561 | 3575 |
| | PAR + VPD + $\theta_{0\text{-}30cm}$ | 0.2182 | 3490 | 3509 |
| | PAR + $\theta_{0\text{-}30cm}$ | 0.2181 | 3488 | 3502 |
| | $Ts_{5cm}$ | 0.2413 | 3462 | 3472 |
| | VPD + $Ts_{5cm}$ | 0.2547 | 3450 | 3464 |
| | $Ts_{5cm}$ + $\theta_{0\text{-}30cm}$ | 0.2552 | 3450 | 3464 |
| | VPD + $Ts_{5cm}$ + $\theta_{0\text{-}30cm}$ | 0.2615 | 3445 | 3464 |
| | PAR + VPD + $Ts_{5cm}$ | 0.3130 | 3387 | 3406 |
| | PAR + $Ts_{5cm}$ | 0.3119 | 3386 | 3400 |
| | PAR + VPD + $Ts_{5cm}$ + $\theta_{0\text{-}30cm}$ | 0.3208 | 3380 | 3403 |
| | PAR + $Ts_{5cm}$ + $\theta_{0\text{-}30cm}$ | 0.3208 | 3378 | 3397 |

| | Model | $R^2$ | AIC | BIC |
|---|---|---|---|---|
| **b) GEP (daily)** | VPD | 0.0245 | 4147 | 4157 |
| | PAR + VPD | 0.1259 | 4062 | 4076 |
| | PAR | 0.1247 | 4061 | 4070 |
| | VPD + $\theta_{0\text{-}30cm}$ | 0.1272 | 4061 | 4075 |
| | $\theta_{0\text{-}30cm}$ | 0.1272 | 4059 | 4068 |
| | PAR + $\theta_{0\text{-}30cm}$ | 0.2150 | 3976 | 3990 |
| | PAR + VPD + $\theta_{0\text{-}30cm}$ | 0.2241 | 3969 | 3987 |
| | $Ts_{5cm}$ | 0.6281 | 3378 | 3387 |
| | VPD + $Ts_{5cm}$ | 0.6330 | 3369 | 3383 |
| | $Ts_{5cm}$ + $\theta_{0\text{-}30cm}$ | 0.6358 | 3363 | 3377 |
| | VPD + $Ts_{5cm}$ + $\theta_{0\text{-}30cm}$ | 0.6376 | 3361 | 3380 |
| | PAR + $Ts_{5cm}$ | 0.6744 | 3274 | 3288 |
| | PAR + $Ts_{5cm}$ + $\theta_{0\text{-}30cm}$ | 0.6860 | 3247 | 3265 |
| | PAR + VPD + $Ts_{5cm}$ | 0.6960 | 3221 | 3240 |
| | PAR + VPD + $Ts_{5cm}$ + $\theta_{0\text{-}30cm}$ | 0.6998 | 3213 | 3236 |

| | Model | $R^2$ | AIC | BIC |
|---|---|---|---|---|
| **c) RE (daily)** | VPD | 0.0027 | 3307 | 3316 |
| | PAR | 0.0049 | 3305 | 3314 |
| | PAR + VPD | 0.0117 | 3301 | 3315 |
| | PAR + $\theta_{0\text{-}30cm}$ | 0.0208 | 3294 | 3308 |
| | $\theta_{0\text{-}30cm}$ | 0.0186 | 3294 | 3303 |
| | VPD + $\theta_{0\text{-}30cm}$ | 0.0338 | 3283 | 3297 |
| | PAR + VPD + $\theta_{0\text{-}30cm}$ | 0.0420 | 3279 | 3297 |
| | $Ts_{5cm}$ | 0.6549 | 2460 | 2469 |
| | PAR + $Ts_{5cm}$ | 0.6606 | 2448 | 2463 |
| | PAR + VPD + $Ts_{5cm}$ | 0.7411 | 2234 | 2253 |
| | VPD + $Ts_{5cm}$ | 0.7407 | 2234 | 2248 |
| | $Ts_{5cm}$ + $\theta_{0\text{-}30cm}$ | 0.7851 | 2084 | 2098 |
| | PAR + $Ts_{5cm}$ + $\theta_{0\text{-}30cm}$ | 0.7869 | 2079 | 2098 |
| | PAR + VPD + $Ts_{5cm}$ + $\theta_{0\text{-}30cm}$ | 0.8171 | 1959 | 1982 |
| | VPD + $Ts_{5cm}$ + $\theta_{0\text{-}30cm}$ | 0.8170 | 1958 | 1976 |

**Table S1.** Rankings of linear regression models for daily sums of: a) non gap-filled net ecosystem productivity (NEP), b) gross ecosystem productivity (GEP), and c) ecosystem respiration from 2012 to 2016 using Akaike Information Criterion (AIC) and Bayesian Information Criterion (BIC) (n = 798).

| | Model | $R^2$ | AIC | BIC |
|---|---|---|---|---|
| **a) NEP (hhour)** | VPD | 0.0172 | -12218 | -12203 |
| | $\theta_{0\text{-}30cm}$ | 0.0789 | -13380 | -13365 |
| | VPD + $\theta_{0\text{-}30cm}$ | 0.0804 | -13409 | -13385 |
| | VPD + $Ts_{5cm}$ | 0.1945 | -15785 | -15762 |
| | $Ts_{5cm}$ | 0.1945 | -15786 | -15771 |
| | VPD + $Ts_{5cm}$ + $\theta_{0\text{-}30cm}$ | 0.1966 | -15830 | -15798 |
| | $Ts_{5cm}$ + $\theta_{0\text{-}30cm}$ | 0.1966 | -15831 | -15808 |
| | PAR | 0.2778 | -17744 | -17728 |
| | PAR + VPD | 0.2917 | -18092 | -18068 |
| | PAR + $\theta_{0\text{-}30cm}$ | 0.3218 | -18869 | -18845 |
| | PAR + VPD + $\theta_{0\text{-}30cm}$ | 0.3586 | -19869 | -19837 |
| | PAR + $Ts_{5cm}$ + $\theta_{0\text{-}30cm}$ | 0.4207 | -21695 | -21664 |
| | PAR + $Ts_{5cm}$ | 0.4207 | -21696 | -21673 |
| | PAR + VPD + $Ts_{5cm}$ | 0.4707 | -23313 | -23282 |
| | PAR + VPD + $Ts_{5cm}$ + $\theta_{0\text{-}30cm}$ | 0.4734 | -23406 | -23367 |

| | Model | $R^2$ | AIC | BIC |
|---|---|---|---|---|
| **b) GEP (hhour)** | VPD | 0.0141 | -9838 | -9822 |
| | $\theta_{0\text{-}30cm}$ | 0.0926 | -11327 | -11312 |
| | VPD + $\theta_{0\text{-}30cm}$ | 0.0930 | -11331 | -11308 |
| | PAR | 0.2500 | -14743 | -14727 |
| | PAR + VPD | 0.2640 | -15081 | -15057 |
| | PAR + $\theta_{0\text{-}30cm}$ | 0.3063 | -16141 | -16117 |
| | PAR + VPD + $\theta_{0\text{-}30cm}$ | 0.3468 | -17219 | -17188 |
| | $Ts_{5cm}$ | 0.3553 | -17458 | -17442 |
| | $Ts_{5cm}$ + $\theta_{0\text{-}30cm}$ | 0.3561 | -17478 | -17454 |
| | VPD + $Ts_{5cm}$ | 0.3582 | -17536 | -17513 |
| | VPD + $Ts_{5cm}$ + $\theta_{0\text{-}30cm}$ | 0.3585 | -17542 | -17511 |
| | PAR + $Ts_{5cm}$ | 0.5391 | -23476 | -23452 |
| | PAR + $Ts_{5cm}$ + $\theta_{0\text{-}30cm}$ | 0.5433 | -23637 | -23606 |
| | PAR + VPD + $Ts_{5cm}$ | 0.6103 | -26484 | -26453 |
| | PAR + VPD + $Ts_{5cm}$ + $\theta_{0\text{-}30cm}$ | 0.6104 | -26487 | -26448 |

| | Model | $R^2$ | AIC | BIC |
|---|---|---|---|---|
| **c) RE (hhour)** | VPD | 0.0023 | -63470 | -63455 |
| | PAR | 0.0031 | -63485 | -63470 |
| | PAR + VPD | 0.0037 | -63495 | -63471 |
| | VPD + $\theta_{0\text{-}30cm}$ | 0.0224 | -63834 | -63811 |
| | $\theta_{0\text{-}30cm}$ | 0.0224 | -63836 | -63821 |
| | PAR + $\theta_{0\text{-}30cm}$ | 0.0237 | -63857 | -63834 |
| | PAR + VPD + $\theta_{0\text{-}30cm}$ | 0.0240 | -63862 | -63831 |
| | $Ts_{5cm}$ | 0.6677 | -83189 | -83174 |
| | PAR + $Ts_{5cm}$ | 0.6699 | -83307 | -83284 |
| | VPD + $Ts_{5cm}$ | 0.7053 | -85343 | -85320 |
| | PAR + VPD + $Ts_{5cm}$ | 0.7070 | -85446 | -85415 |
| | $Ts_{5cm}$ + $\theta_{0\text{-}30cm}$ | 0.7965 | -91988 | -91964 |
| | PAR + $Ts_{5cm}$ + $\theta_{0\text{-}30cm}$ | 0.7968 | -92010 | -91979 |
| | VPD + $Ts_{5cm}$ + $\theta_{0\text{-}30cm}$ | 0.8103 | -93242 | -93211 |
| | PAR + VPD + $Ts_{5cm}$ + $\theta_{0\text{-}30cm}$ | 0.8117 | -93371 | -93332 |

**Table S2.** Rankings of linear regression models for half-hourly sums of: a) non gap-filled net ecosystem productivity (NEP), b) gross ecosystem productivity (GEP), and c) ecosystem respiration from 2012 to 2016 using Akaike Information Criterion (AIC) and Bayesian Information Criterion (BIC) (n = 17,937).
