# Supplementary figures and images for: The Impact of Seasonal and Annual Climate Variations on the Carbon Uptake Capacity of a Deciduous Forest Within the Great Lakes Region of Canada
Source: J Geophys Res Biogeosci. 2020 Sep 18;125(9):e2019JG005389. doi: 10.1029/2019JG005389 (PMC7540005; doi:10.1029/2019JG005389)

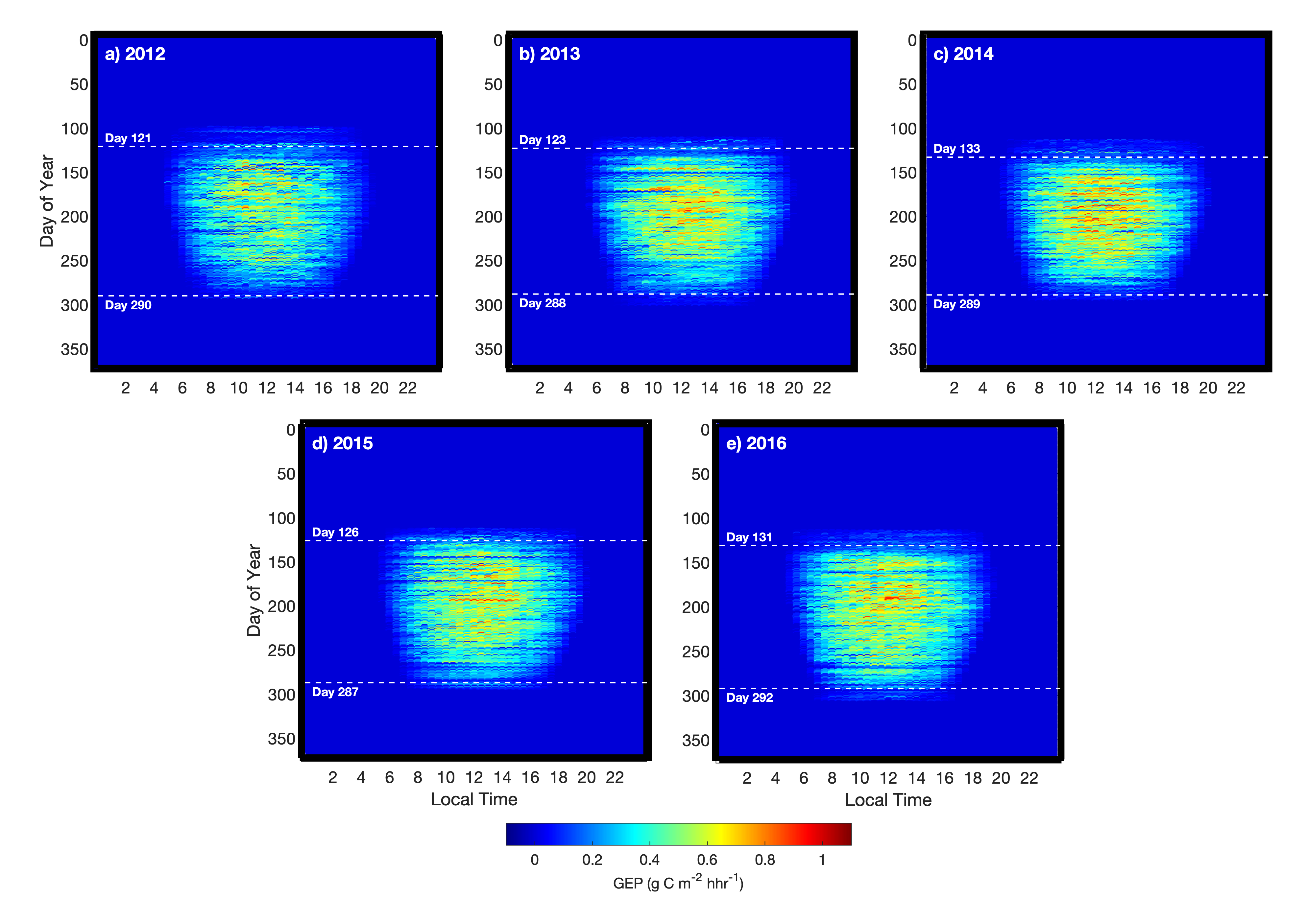

Supplement: Supplementary file 2 — Figure S1 [file JGRG-125-e2019JG005389-s002.tif]

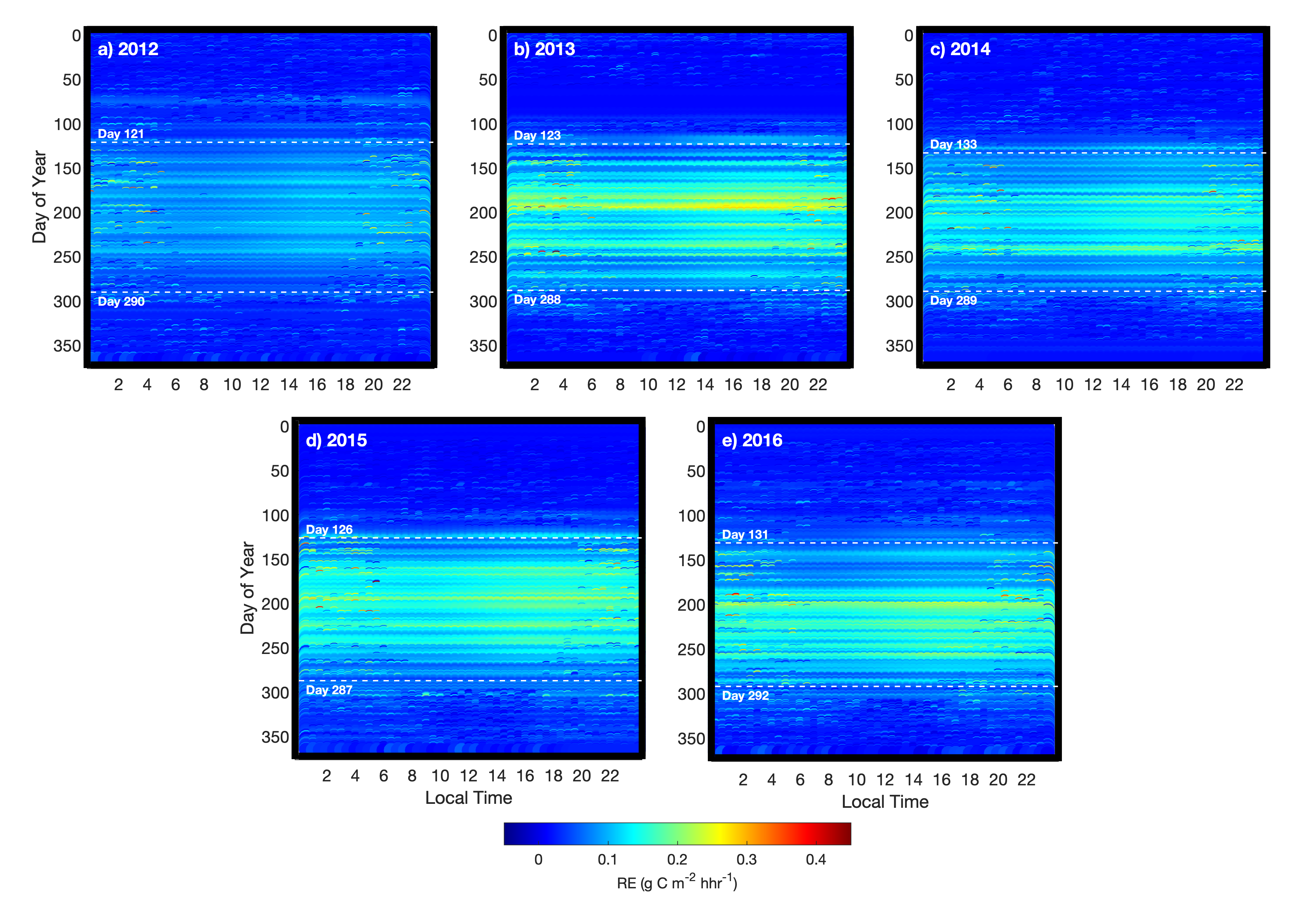

Supplement: Supplementary file 3 — Figure S2 [file JGRG-125-e2019JG005389-s003.tif]
